# How Visual Word Decoding and Context-Driven Auditory Semantic Integration Contribute to Reading Comprehension: A Test of Additive vs. Multiplicative Models

**DOI:** 10.3390/brainsci11070830

**Published:** 2021-06-23

**Authors:** Yu Li, Hongbing Xing, Linjun Zhang, Hua Shu, Yang Zhang

**Affiliations:** 1 Division of Science and Technology, BNU-HKBU United International College, Zhuhai 519087, China; yuli@uic.edu.cn; 2 Institute on Education Policy and Evaluation of International Students, Beijing Language and Culture University, Beijing 100083, China; xinghb@blcu.edu.cn; 3 Beijing Advanced Innovation Center for Language Resources and College of Advanced Chinese Training, Beijing Language and Culture University, Beijing 100083, China; 4 State Key Laboratory of Cognitive Neuroscience and Learning, Beijing Normal University, Beijing 100875, China; shuh@bnu.edu.cn; 5 Department of Speech-Language-Hearing Sciences and Center for Neurobehavioral Development, University of Minnesota, Minneapolis, MN 55455, USA

**Keywords:** reading comprehension, speech-in-noise recognition, nature *F*_0_ contours, flattened *F*_0_ contours, Chinese character decoding

## Abstract

Theories of reading comprehension emphasize decoding and listening comprehension as two essential components. The current study aimed to investigate how Chinese character decoding and context-driven auditory semantic integration contribute to reading comprehension in Chinese middle school students. Seventy-five middle school students were tested. Context-driven auditory semantic integration was assessed with speech-in-noise tests in which the fundamental frequency (*F*_0_) contours of spoken sentences were either kept natural or acoustically flattened, with the latter requiring a higher degree of contextual information. Statistical modeling with hierarchical regression was conducted to examine the contributions of Chinese character decoding and context-driven auditory semantic integration to reading comprehension. Performance in Chinese character decoding and auditory semantic integration scores with the flattened (but not natural) *F*_0_ sentences significantly predicted reading comprehension. Furthermore, the contributions of these two factors to reading comprehension were better fitted with an additive model instead of a multiplicative model. These findings indicate that reading comprehension in middle schoolers is associated with not only character decoding but also the listening ability to make better use of the sentential context for semantic integration in a severely degraded speech-in-noise condition. The results add to our better understanding of the multi-faceted reading comprehension in children. Future research could further address the age-dependent development and maturation of reading skills by examining and controlling other important cognitive variables, and apply neuroimaging techniques such as functional magmatic resonance imaging and electrophysiology to reveal the neural substrates and neural oscillatory patterns for the contribution of auditory semantic integration and the observed additive model to reading comprehension.

## 1. Introduction

Reading comprehension involves the construction of literal and inferred meanings from linguistic input in print, which requires cognitive operations at lower-level processes of visual feature extraction and word decoding as well as higher-level processes of lexical access, syntactic analysis, and semantic integration. While different levels of cognitive and linguistic processing might have unique contributions to reading comprehension [1,2,3,4,5], these processes can be simplified to two separate components, i.e., decoding (recognizing printed words) and linguistic comprehension (taking lexical information and deriving sentence and discourse interpretations with listening comprehension most often measured as a substitute) [1,6]. Specifically, the Simple View of Reading (SVR) postulates that once isolated printed words are recognized, the remaining processes in reading comprehension are analogous to those involved in listening comprehension. At the neural level, functional magnetic resonance imaging studies have revealed that in addition to modality-specific brain activation, the two modalities of language comprehension activate common brain regions of the temporal and inferior frontal cortices associated with semantic processing [7,8,9], thus providing neural evidence for shared cognitive processes underlying reading and listening comprehension. Of these cognitive processes, semantic integration is an essential bridging element by which listeners integrate lexical–semantic information with pragmatic and sociolinguistic knowledge to reach an interpretation that reflects semantic representations [10]. Given the critical role of auditory semantic integration in the context of discourses in listening comprehension, the abilities to recognize printed words and make use of top-down contextual semantic information could serve as good predictors for reading comprehension.

During speech recognition and understanding, top-down semantic integration is particularly important in adverse conditions in which acoustic–phonetic analysis alone is insufficient for the identification of particular words because of suboptimal listening backgrounds and/or poor speech signal quality. Specifically, the presence of various types of interference such as broadband noise and one-/multi-talker babbles deteriorates speech intelligibility dramatically, but listeners are able to use semantic context to offset the detrimental effects to a great extent [11,12,13,14]. Similarly, semantic context is also used by listeners to aid speech recognition and comprehension when the speech signal itself is degraded [15,16]. Furthermore, when a degraded speech signal is presented against interference, listeners benefit even more from semantic context. For example, the intelligibility difference between acoustically degraded sentences and semantically unrelated words is much greater when they are presented in suboptimal listening backgrounds than in quiet, indicating that listeners rely more on the top-down semantic context to aid speech recognition and comprehension in adverse conditions [16,17,18].

The development of the auditory semantic integration ability to help speech recognition and comprehension occurs during early age and continues to improve over the entire childhood. While children are able to use semantic context to assist speech recognition in quiet as early as 2 years old [19], the ability to use semantic context in adverse listening backgrounds continues to improve into adolescence. Previous studies on speech intelligibility of high- and low-predictability sentences in children at 5–17 years of age consistently showed that contextual semantic cues assisted children of all ages in identifying words masked by babbles, with older children performing better than younger children in the high-context conditions [20,21,22]. For example, elementary school children aged 11 and 13 years performed significantly poorer than middle school children aged 15 and 17 years on speech recognition in noise, and this difference occurred primarily for high-predictability sentences presented at the 0 dB signal-to-noise (SNR) ratio [20]. In addition, there are developmental changes in children’s ability to use semantic context to decode degraded signals in quiet and interfering backgrounds [23,24]. For example, children aged 5–7 years required as much acoustic–phonetic information with high-predictability as with low-predictability contexts to identify target words presented in quiet. By contrast, children aged 8–10 years required shorter portions of the target words (i.e., less acoustic–phonetic information) for recognition in high-predictability than in low-predictability sentences [24]. Our previous study [25] showed that elementary school children aged 10 years could not make better use of semantic context in recognizing speech with flattened fundamental frequency (*F*_0_) contours compared to speech with natural *F*_0_ contours. By contrast, middle school children aged 14 years benefited more from semantic context when natural *F*_0_ contours were altered, regardless of whether the spoken sentences were presented in quiet or suboptimal listening backgrounds. These findings suggest that semantic context can be better used to facilitate listening comprehension and speech recognition by middle school students compared with younger children.

Reading is a cultivated process that maps written symbols to phonological and semantic representations, and learning to read universally involves solid and stable interactions between written and spoken languages through a large amount of training in schools or other learning experiences. Chinese has a logographic writing system, which is markedly different from alphabetic languages. In alphabetic languages, graphemes that correspond to phonemes of spoken languages are used as visual symbols and word decoding follows grapheme-to-phoneme conversion rules. In written Chinese, however, characters are used as basic writing units with no parts in a character corresponding to phonemes. It is never the case in the Chinese writing system that a phonetic component maps onto a sub-syllabic phonological representation in the way that a letter maps onto a part of a word’s phonological form in an alphabetic system [26]. Furthermore, the layout of graphemes in an alphabetic language is linear, whereas a Chinese character is composed of intricate strokes packed into a square configuration. Full literacy in Chinese requires knowledge of between 3000 and 4000 different characters and it relies to a large degree on rote memory, which is in sharp contrast to that of alphabetic languages which use a relatively limited number of symbols (typically 22–30 or so letters of the alphabet) to produce all words in the languages [27]. Therefore, learning to read can present a much greater challenge for Chinese-speaking children than for children who speak alphabetic languages, and there is evidence that the challenge persists into the late stage of reading development. It takes children a longer time to achieve decoding accuracy when learning to read in a deep orthography compared with a shallow orthography [28]. Moreover, studies on alphabetic languages have revealed that the relation between decoding and reading comprehension decreases sharply after age 9–10 years, indicating a developmental transition [29,30]. Considering the opaqueness and visual complexity of Chinese orthography, the transition may be much delayed, and probably occurs after elementary schooling. Indeed, previous studies of Chinese reading have suggested that decoding is still a strong predictor of reading comprehension in middle school children aged 13–14 years [31,32,33]. The correlation coefficients (0.55–0.70) reported in these studies are higher than those found in English-speaking children with similar age (0.41 in [30]). However, during the development of Chinese reading ability, it remains unclear how the processes of character decoding and contextual semantic integration for speech recognition may independently or interactively contribute to reading comprehension. This critical issue is particularly pertinent to the middle school students who have already got through the early stage of learning to read with a sizable vocabulary [34] but are still facing the challenge of reading development in Chinese.

Based on these extant studies on the development of auditory semantic integration ability and character decoding and the SVR theory, it can be predicted that these two abilities can serve as good predictors of reading comprehension in Chinese middle school children. However, to our knowledge, no previous studies have investigated this issue systematically. This model-testing study aimed to fill this gap by examining the relative contributions of the two subskills to reading comprehension. The Chinese language is particularly interesting and suitable for our purpose due to its special orthographic system that takes considerable amounts of time to learn and a phonological system that deploys pitch contour variations at each syllable (known as the lexical tones) for different words. Specifically, to assess the role of contextual semantic integration in speech recognition, we introduced acoustic manipulations in two speech-in-noise conditions, one with natural *F*_0_ contours kept in the target sentences presented against interfering background speech, and the other with flattened *F*_0_ contours that disrupted the critical cue for Chinese lexical tones for proper word recognition. This speech-in-noise test protocol with Mandarin Chinese materials was previously adopted in a number of studies on elementary school students, middle school students, and adults, including the elderly population [16,18,25], and the results demonstrate that greater auditory semantic integration at the sentence level is required to recognize the words in the *F*_0_-degraded condition. Furthermore, two statistical models, i.e., the multiplicative model (product of the two subskills) and additive model (sum of the two subskills), were tested to clarify how the subskills would predict the variance in reading comprehension. The multiplicative model predicts that if an individual performs poorly on one subskill, reading comprehension would also be poor no matter how he or she performs on the other subskill. The additive model predicts that reading comprehension is adequately explained by the linear combination of the two subskills. There has been mounting evidence from studies of reading comprehension that the additive model of decoding and linguistic comprehension fitted data well and could provide a better fit than the multiplicative model of the two subskills, and beyond the additive model, the multiplicative model did not provide further significant contribution to reading comprehension in alphabetic languages [35,36,37] and Chinese [38]. Thus, we hypothesized that the additive model might provide better predictive modeling results for our data. The findings of the present study will add to our understanding of the cognitive model of reading to further delineate the developmental trajectory of reading skills, which can provide the foundation to examine the brain mechanisms underlying the acquisition of normal reading skills in children as well as the disruptive neural correlates accompanying developmental dyslexia [39,40,41,42,43,44].

## 2. Methods and Materials

### 2.1. Participants

Seventy-five middle school students (37 females; mean age, 14.2 years; age range, 13.25 to 15.44 years; 59 Grade 8, 16 Grade 9) were recruited from a middle school located in Beijing to participate in this study. Each had normal or corrected-to-normal vision, and had no known hearing impairments or history of neurological disorders. Participation was voluntary with consent obtained from their legal guardians. This study was approved by the Institute Review Board of Beijing Normal University in compliance with the Declaration of Helsinki for the protection of human subjects.

### 2.2. Test Protocols and Measures

Three independent tests were carried out, measuring Chinese character decoding, semantic integration during speech recognition against interfering background speech, and reading comprehension, respectively. The children were individually tested in quiet rooms with ambient noise level below 45 dB(A). Details of these test procedures are described below.

### 2.3. Chinese Character Decoding Test

The test that was used to estimate children’s decoding ability consisted of 150 characters. The first 40 items were taken from a Chinese character decoding test for kindergarten children [45]. One hundred of the remaining 110 characters were taken from Chinese language textbooks for elementary school children with 20 items from each grade level from Grades 2 to 6 [46]. The last 10 characters had not been introduced in textbooks for both kindergarten and elementary school children. There were 42 regular phonetic characters, 62 irregular phonetic characters, and 46 non-phonetic characters in the test. Of these characters, low-frequency (less than 10 per million) characters accounted for 15.3%, i.e., 23 characters, intermediate-frequency (between 10 and 100 per million) characters 48.7%, i.e., 73 characters, and high-frequency (above 100 per million) characters 36%, i.e., 54 characters. Four Mandarin tones were all covered in the test. The number of strokes in a character ranged from 2 to 19 and the mean stroke number was 10.2. During the test, children were asked to read the characters aloud one by one and the testing was terminated after reading 15 consecutive items incorrectly. Each character was worth one mark. The performance score was the number of correctly pronounced characters. This test was widely used in our previous studies of Chinese child reading and demonstrated excellent reliability and validity [40,47,48].

### 2.4. Auditory Semantic Integration during Speech Recognition against Interference

**Figure 1:**
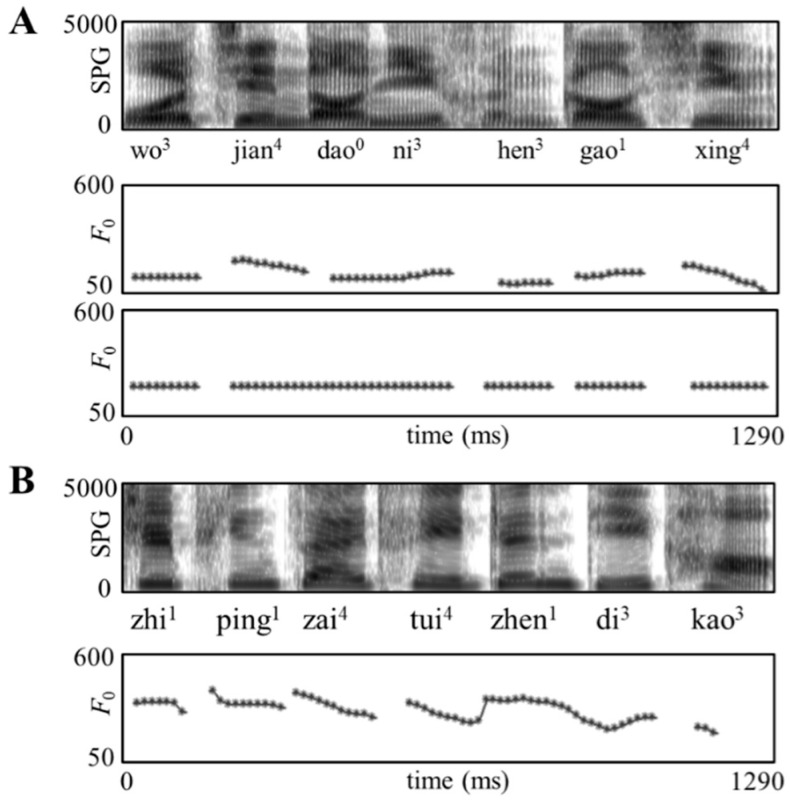
Acoustic features of sample speech stimuli. (**A**) Broadband spectrograms (SPG: 0 to 5 kHz) and fundamental frequency (*F*_0_: 50 to 600 Hz) contours are displayed for a sentence with natural *F*_0_ contours and its pitch-flattened counterpart; (**B**) An interfering sentence with natural *F*_0_ contours. Note: The samples are transcribed in Pinyin (the official Romanization system for Standard Chinese) and the figures in the upper right corners signify lexical tones.

Performance in speech recognition against interference was used to indicate auditory semantic integration ability. Stimuli in this speech recognition test were taken from a corpus used in our previous study [25] and only a brief description is provided here. In this speech-in-noise test, two types of sentences, i.e., sentences with natural and flattened *F*_0_ contours, were used as targets. Sentences with natural *F*_0_ contours were read by a male native Chinese speaker and the monotonous sentences with flat *F*_0_ contours were created by flattening the natural *F*_0_ contours at each sentence’s mean *F*_0_. Masker stimuli were consonant-misplaced sentences, which were constructed based on the normal sentences. Specifically, the initial consonant of every syllable in each normal sentence was replaced with another consonant as long as the phonotactic rules of Chinese were not violated. Consonant-misplaced sentences were unintelligible at both word and sentence levels, thus having minimal effect of informational masking. For example, the consonant-misplaced sentence “zhi^1^ ping^1^ zai^4^ tui^4^ zhen^1^ di^3^ kao^3^” is constructed based on the normal sentence “chi^1^ qing^1^ cai^4^ dui^4^ shen^1^ ti^3^ hao^3^ (Eating vegetables is good for health)”. A female native Mandarin speaker read the masker sentences, which was to enable easy separation of the target message read by a male from the interfering speech (Figure 1). The target and masker sentences were first edited to be at sound pressure levels of 75 and 70 dB, respectively, and then combined to form the speech against interference stimuli with SNR level set at +5 dB (refer to the supplementary materials for the sample stimuli).

The speech stimuli were delivered via a pair of Edifier R18 loudspeakers with the sound level set at 65 dB SPL calibrated at the approximate center position of the listener’s head. The participants were instructed to listen to the target sentences in the male voice carefully and verbally repeat what they heard for the sentences with natural *F*_0_ contours and what the sentences should be for the sentences with natural *F*_0_ contours, as commonly administered in a typical speech intelligibility test. Before the actual test phase, all children participated in a brief practice session representing samples of the experimental conditions. This test protocol adopted a self-paced paradigm, and the participants were encouraged to take a guess when they were not sure which words they had heard. Each child was presented with a total of 28 sentences, half with natural *F*_0_ contours and the other half with flat *F*_0_ contours. The speech materials were prepared to ensure that each participant did not listen to the same sentence with natural or flat *F*_0_ contours and the order of *F*_0_ patterns was counterbalanced across the participants. The responses were recorded and scored by the first author of this paper and checked by an independent auditor blind to the experiment. A strict score standard was adopted. Specifically, only words with consonants, vowels, and lexical tones all correctly identified were considered correct answers. The performance score was the proportion of the number of correctly reported words, which was then converted to rationalized arcsine transform units (RAU) [49] for the final statistical analyses. The use of RAU transformation helps to reduce ceiling-level saturation and restore the homoscedasticity required of normal distribution for parametric statistical tests, as the distribution of the scores, i.e., proportion data, was not strictly Gaussian.

### 2.5. Reading Comprehension Test

The test, developed by following the procedure in Moll, Fussenegger, Willburger, and Landerl [50], consists of 100 sentences or short passages with the number of characters (including punctuations) in each item gradually increasing from 7 to 159. The first 90 items were from our early work (Lei et al., 2011) and the last 10 items were developed afterwards. There were more than 3000 characters contained in all items and all participants were familiar with the characters and words. Of these 100 items, 50 items were semantically correct. Semantically incorrect sentences were made by contradicting inferences from the sentences with world knowledge or knowledge of syntax (e.g., the sun rises from the west every day). In the task, each child was given 3 min to read as many items as possible and indicate whether they were semantically correct or not using ‘√’ or ‘×’ symbol at the end of each item. Three examples were given to ensure understanding of the task before the actual test. The performance score was the number of correctly judged items. Excellent reliability and validity were demonstrated in our previous work on children with dyslexia and typically developing children [40,47,51].

### 2.6. Data Analysis

Partial correlation analyses after controlling for age and sex were first carried out to examine the relationship between different measures. Hierarchical regression analyses were then conducted to extract the relative contributions of character decoding and auditory semantic integration to reading comprehension, with different orders of the performance in the tests entered into the analyses and the relative contributions of the additive model and multiplicative model. The additive model was defined as the total variance of character decoding and speech recognition no matter what order entered into the hierarchical regression analyses was used, and the multiplicative model as the variance of the multiplication of the two factors (see [52] for the same approach).

## 3. Results

Performance scores in the Chinese character decoding, speech recognition against interference, and reading comprehension are summarized in Table 1. A paired-samples *t*-test analysis revealed that recognition of speech with normal *F*_0_ contours was significantly better than that of speech with flattened *F*_0_ contours (t(74) = 9.69, *p* < 0.001), indicating that the latter was more difficult for the listeners.

Table 2 depicts the partial correlations between different measures after controlling for age and sex. The results show that both Chinese character decoding and reading comprehension were positively correlated with recognition of speech with flattened *F*_0_ contours, but not significantly correlated with recognition of speech with normal *F*_0_ contours. Accuracy scores on the two speech recognition measures were positively correlated.

Hierarchical regression analyses further revealed that Chinese character decoding significantly accounted for the most variance in reading comprehension when it was entered before recognition of speech with normal *F*_0_ contours (13.1%), and the contribution decreased slightly (11.7%) when speech recognition was entered before character decoding. Recognition of speech with normal *F*_0_ contours did not significantly contribute to the variance in reading comprehension no matter if it was entered before or after Chinese character decoding (Table 3A,B). However, both Chinese character decoding and recognition of speech with flattened *F*_0_ contours significantly or marginally significantly accounted for the variance in reading comprehension, with either of them contributing more when first entered (Table 4A,B).

The hierarchical regression analyses also revealed that the additive model contributed to 14.0% of the variance in reading comprehension for recognition of speech with normal *F*_0_ contours and the percentage increased to 16.3% for recognition of speech with flattened *F*_0_ contours. By comparison, the multiplicative model contributed to 10.6% of the variance in reading comprehension for recognition of speech with normal *F*_0_ contours and the percentage increased to 14.8% for recognition of speech with flattened *F*_0_ contours (Table 3C and Table 4C). These results indicate that the additive model accounted for more variance than the multiplicative model.

## 4. Discussion

Reading comprehension encompasses various components and the past decades have witnessed great progress in the understanding of its complexity. Based on the Simple View of Reading (SVR) framework, the present study examined the contribution of Chinese character decoding and auditory semantic integration to reading comprehension among Chinese middle school students who had largely passed the early stage of learning to read during elementary schooling and were at the stage of reading to learn new knowledge [34]. The results show that Chinese character decoding significantly contributed to reading comprehension irrespective of the extent of auditory semantic integration, indicating that children who know a greater amount of characters have better reading comprehension. Recognition of speech with natural *F*_0_ contours did not significantly contribute to reading comprehension; however, a significant contribution was observed when natural *F*_0_ contours were flattened, indicating that children who can make better use of sentence-level contextual semantic integration in a more adverse condition have better reading ability. Furthermore, based on the predictive power results, the contributions of the two subskills to reading comprehension fitted better with the additive model than the multiplicative model. By examining the contribution of auditory semantic integration to reading, the current study highlights the complexity of reading comprehension.

Because of the lack of clear grapheme-to-phoneme mapping and complex layout of characters in the Chinese logographic writing system, the number of recognized characters plays a critical role in early reading development. For example, Joshi, Tao, Aaron, and Quiroz [53] found that the performance in Chinese character decoding explained 22% of the variance in reading comprehension among Grade 2 (about 8 years old) children and 32% in Grade 4 (about 10 years old) children. Together with their findings, the results of the current study confirm that Chinese character decoding contributed significantly to reading comprehension for middle school students, which suggests that the role of Chinese character decoding persists throughout the reading development process before maturity. Because different materials were adopted for the measurement of Chinese character decoding and reading comprehension in the current study and Joshi et al.’s study, it is impossible to make direct comparisons between the contributions of character decoding to reading comprehension and obtain the developmental trend from elementary school to middle school in children. Chung et al. [31,32,33] recently found high correlations (*r*s > 0.50) between Chinese character decoding and reading comprehension in middle school children. These findings indicate that it is still hard to establish a developmental trend in Chinese, at least not in elementary and middle school children. Studies in alphabetic languages, however, indicate an overall trend in which the importance of word decoding in reading comprehension decreases from elementary school to middle school [30]. Further investigations are needed to explore the developmental trend in the Chinese logographic writing system. It is noteworthy that Chinese character decoding ability can be measured with both accuracy and fluency. In the current study, only accuracy scores were obtained. There has been some evidence that Chinese character decoding fluency contributes to reading comprehension in elementary school children [53,54]. Because middle school children are at the intermediate stage from premature readers to mature readers [34], future longitudinal studies need to examine which measure is a stronger predictor of reading comprehension.

As auditory semantic integration in the sentential context is essential to listening comprehension [10], we predicted that it could serve as a good predictor for reading comprehension. In the present study, the extent of semantic integration needed for recognizing the target spoken sentences was manipulated by presenting two types of stimuli. Specifically, in suboptimal listening conditions, greater auditory semantic integration is required for successful recognition of speech with flattened *F*_0_ contours compared with speech with nature *F*_0_ contours [16,18]. Interestingly, our results show that recognition of speech with flattened *F*_0_ contours contributed to reading comprehension, while the contribution decreased dramatically when the natural *F*_0_ contours in the target spoken sentences were intact. These results indicate that the more auditory semantic integration is involved in a speech recognition task, the more the listening task contributes to reading comprehension. There are developmental changes for children to use semantic context to aid speech recognition in adverse conditions. For example, our previous study showed that middle school children could make better use of semantic context in recognizing speech with flattened *F*_0_ contours compared to speech with natural *F*_0_ contours, but elementary school children could not, although they could utilize semantic context when recognizing speech with natural *F*_0_ contours [25]. How the ability to make use of semantic information to aid recognition of speech with natural *F*_0_ contours contributes to reading comprehension among elementary school children needs further investigation.

Regarding the statistical modeling issue of how word decoding and linguistic comprehension combine to explain the variance in reading comprehension, previous studies have found that the additive model performed as well as or better than the multiplicative model in explaining the variance in reading comprehension [35,36,37]. However, this issue is still rarely examined in Chinese. A recent study on Chinese reading comprehension among elementary school children revealed that, overall, the multiplicative model did not contribute a significant amount of variance to reading comprehension in addition to the additive model [38]. Although our findings are not meant to be directly compared with those of the previous studies because different subskills were measured, all these studies collectively provide evidence in support of the additive model from a general perspective. Interestingly, the respective contributions of character decoding and recognition of speech with flattened *F*_0_ contours to reading comprehension depended on which one was first entered. When speech recognition was first entered, the relative contribution of character decoding was still significant but much reduced, and vice versa. These results echo the multiplicative model, showing a higher contribution of the two components than either of them. However, similar patterns were not observed for the recognition of speech with natural *F*_0_ contours. When the *F*_0_ contours are flattened, speech recognition depends more on the semantic context, which has been demonstrated in our previous studies covering both children and adults [16,25]. In particular, older children (middle-school age) have been shown to be more adult-like, and benefit more from the semantic context than younger ones when the *F*_0_ contours are flattened. In the current study, the flattened *F*_0_ condition appears to be better suited than the natural *F*_0_ condition to tap into the top-down processing mode of semantic integration, revealing the significant association between visual word decoding and semantic integration for speech comprehension in the auditory modality. There may be additional common mechanisms and cognitive resources that mediate the presence or absence of a significant association between word decoding and auditory comprehension. Uncovering these mediating cognitive processes is beyond the scope of the current investigation, and we hope to clarify them in future studies.

Although the present study specifically examined the relative contributions of Chinese character decoding and auditory semantic integration in adverse conditions to reading comprehension in middle school students, it is limited in several aspects. Firstly, our study was narrowly focused on testing the multiplicative vs. additive models based on the theoretical framework of SVR. We measured only Chinese character decoding and auditory semantic integration abilities but did not measure other reading-related linguistic subskills (e.g., phonological awareness and vocabulary) and cognitive competence (e.g., non-verbal IQ and executive function) that also contribute to reading comprehension. The relative contributions of these interrelated linguistic and cognitive subskills to reading comprehension in Chinese middle school students were examined in Chinese primary school students [38,55,56] and Chinese middle school children [31,32,33]. These findings extended the original version of SVR on which we framed the current experimental design, adding to the better understanding of the complexity of reading comprehension. Further investigations are needed to understand how auditory semantic integration and character decoding contribute to reading comprehension after controlling for these linguistic or cognitive subskills with a more sophisticated multi-level statistical modeling approach, including both fixed and random (such as test item and participant) factors. Secondly, reading comprehension can be measured at various levels such as sentence, paragraph, and passage. Long paragraph and passage comprehension require greater semantic integration than sentence or short passage comprehension used in the present study, which might increase the contribution of auditory semantic integration during speech-in-noise recognition. This issue needs further investigation.

The last two decades have witnessed fast development in neuroimaging techniques such as event-related potentials and magnetic resonance imaging and the application of these techniques in revealing neural correlates of cognitive processing. Previous research has shown that the left posterior fusiform gyrus is much involved in visual word form processing after the basic visual processing [39,57] and that listening and reading comprehension is supported by widely distributed brain regions in the temporal and inferior frontal cortices [7,8,9]. How these regions work in concert to efficiently support the reading processes from visual word form analysis to semantic integration is still an open question [42,43]. Neuroimaging techniques can be employed to reveal and characterize functional and structural connections between the posterior fusiform gyrus and the distributed temporal and frontal regions in typically developing children [58], and thus could serve as a powerful tool to provide neural evidence for the additive model of reading comprehension revealed here. Neuroimaging investigations may also help to reveal how the disruption of these functional and structural connections is associated with aberrant reading behaviors in children with dyslexia who are widely thought to have word reading deficits [41,59]. Furthermore, cognitive neuroscience research has revealed that neural oscillations play an important role in language processing [60,61,62]. For example, it has been shown that distinct neural oscillatory activities are involved in speech comprehension [60,63,64,65], semantic integration for sentence reading [66], and story reading [67]. Future studies could investigate neural oscillatory patterns in association with the findings of the present study, specifically by examining neural oscillation evidence for how visual word decoding and auditory semantic integration jointly contribute to reading comprehension.

## 5. Conclusions

In conclusion, our findings reveal that in Chinese middle school students, Chinese character decoding and auditory semantic integration during speech-in-noise recognition contributed to reading comprehension in an additive manner. The current study emphasizes that in addition to decoding and linguistic comprehension measures that are traditionally thought to be reliable predictors of reading comprehension, children’s performance in speech recognition under some adverse conditions can also serve as a predictor of reading comprehension, because a high degree of semantic integration is involved in these conditions. These new findings from middle school students enrich our understanding of the complexity and decomposition of reading comprehension from a developmental perspective, which supports the additive model for conducting further cognitive brain research with potential clinical implications for intervention with dyslexia.

## Figures and Tables

**Table 1:** Means, ranges, and standard deviations of each measure.

Measures	M	Range	SD
Chinese character decoding	137	118–148	7.07
Recognition of speech with normal *F*_0_ contours	0.93	0.31–1	0.12
Recognition of speech with flattened *F*_0_ contours	0.80	0.23–1	0.14
Reading comprehension	74.51	46–91	9.57

**Table 2:** Correlations between variables after controlling for age and sex.

	1	2	3	4
1. Chinese character decoding	-			
2. Recognition of speech with normal *F*_0_ contours ^a^	0.126	-		
3. Recognition of speech with flattened *F*_0_ contours ^a^	0.362 **	0.547 ***	-	
4. Reading comprehension	0.384 ***	0.182	0.39 **	-

Note. ^a^, transformed data. ** *p* < 0.01. *** *p* < 0.001.

**Table 3:** Hierarchical regression analyses predicting reading comprehension from Chinese character decoding and recognition of speech with normal *F*_0_ contours.

		Reading Comprehension	
**Step**	**Variables**	***R*^2^**	**Δ*R*^2^**
1	Age	0.112	0.112 *
	Sex		
**A**			
2	Chinese character decoding	0.243	0.131 ***
3	Recognition of speech with normal *F*_0_ contours ^a^	0.252	0.009
**B**			
2	Recognition of speech with normal *F*_0_ contours ^a^	0.135	0.023
3	Chinese character decoding	0.252	0.117 ***
**C**			
2	Chinese character decoding × Recognition of speech with normal *F*_0_ contours ^a^	0.219	0.106 **

Note. ^a^, transformed data. * *p* < 0.05. ** *p* < 0.01. *** *p* < 0.001.

**Table 4:** Hierarchical regression analyses predicting reading comprehension from Chinese character decoding and recognition of speech with flattened *F*_0_ contours.

		Reading Comprehension	
**Step**	**Variables**	***R*^2^**	**Δ*R*^2^**
1	Age	0.112	0.112 *
	Sex		
**A**			
2	Chinese character decoding	0.243	0.131 ***
3	Recognition of speech with flattened *F*_0_ contours ^a^	0.275	0.032 ^Ψ^
**B**			
2	Recognition of speech with flattened *F*_0_ contours ^a^	0.200	0.088 **
3	Chinese character decoding	0.275	0.074 **
**C**			
2	Chinese character decoding × Recognition of speech with flattened *F*_0_ contours ^a^	0.261	0.148 ***

Note. ^a^, transformed data. ^Ψ^ *p* < 0.09. * *p* < 0.05. ** *p* < 0.01. *** *p* < 0.001.

## Data Availability

The summary report of original data is included in the article; further inquiries of raw data can be directed to the corresponding authors.
